# Thrombotic ST-segment elevation myocardial infarction caused by a caseous mitral annular calcification

**DOI:** 10.1007/s00392-023-02167-7

**Published:** 2023-02-21

**Authors:** Baravan Al-Kassou, Marcel Weber, Georg Nickenig, Sebastian Zimmer

**Affiliations:** Heart Center Bonn, Department of Medicine II, University Hospital Bonn, Venusberg-Campus 1, 53127 Bonn, Germany

Sirs:

A 73-year-old patient was admitted to the emergency department for an ST-segment elevation myocardial infarction. The patient reported sudden onset of persisting chest pain and dyspnea for three hours. The clinical history of the patient included diabetes, arterial hypertension, and prior stroke in the year 2002.

Initial 12-lead electrocardiogram (ECG) showed sinus rhythm with posterior and inferior ST-segment elevation. The transthoracic echocardiography revealed inferior and lateral hypokinesia of the left ventricle. Moreover, a large, echo-dense mass with central echolucency and well-defined calcified margins was detectable.

Emergency coronary angiography via transradial approach revealed a thrombus in the proximal left circumflex artery. Except for the proximal embolic occlusion, adequate blood flow was detectable in the distal artery. Furthermore, the coronary arteries were free of atheromatous lesions. After thrombus resolution with heparin and tirofiban, recovery of ST segment elevation and improvement in clinical symptoms were observed.

Diagnostic work-up for differentiation of the cardiac tumor included transesophageal echocardiography (TEE), that showed a well-circumscribed, smooth-bordered echo-dense mass (2.9 × 1.9 cm) at the posterior peri-annular region of the mitral valve, containing central echolucency and suspicious for a caseous mitral annular calcification (CMAC, Fig. 1A–C). The color flow Doppler detected a mild mitral regurgitation with a mean pressure gradient of 3.0 mmHg (Fig. 1D). The subsequent cardiac magnetic resonance revealed a calcified caseous formation of the mitral valve annulus adjacent to the posterior mitral valve leaflet, that was hypointense in both T1- and T2-weighted sequences without early or late contrast enhancement, confirming the suspected diagnosis of CMAC (Fig. 2A–B).

**Fig. 1:**
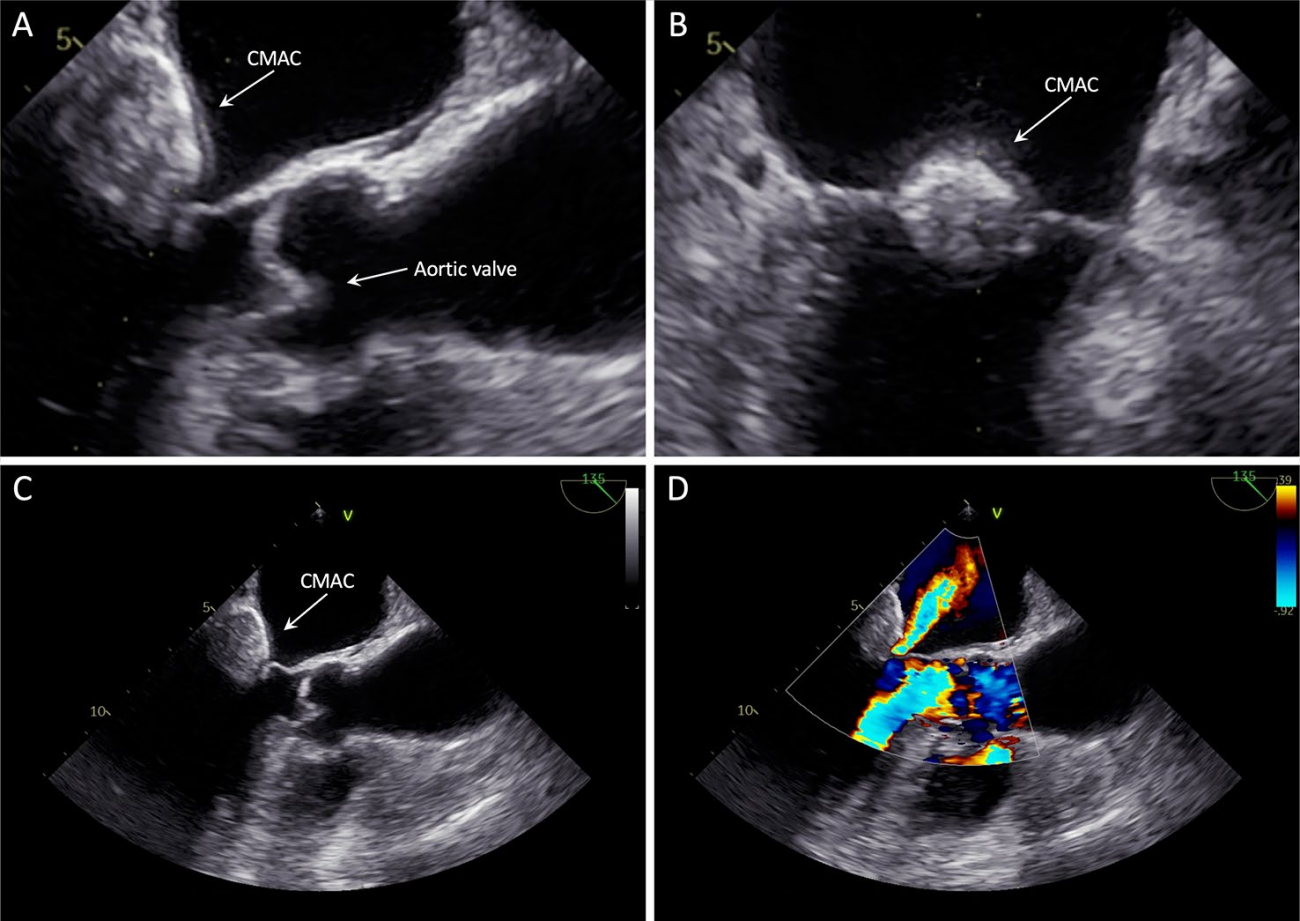
Transesophageal echocardiography showing a well-defined, smooth-bordered echo-dense mass with central echolucency at the posterior peri-annular region of the mitral valve causing a mild mitral regurgitation. *CMAC* caseous mitral annular calcification

**Fig. 2:**
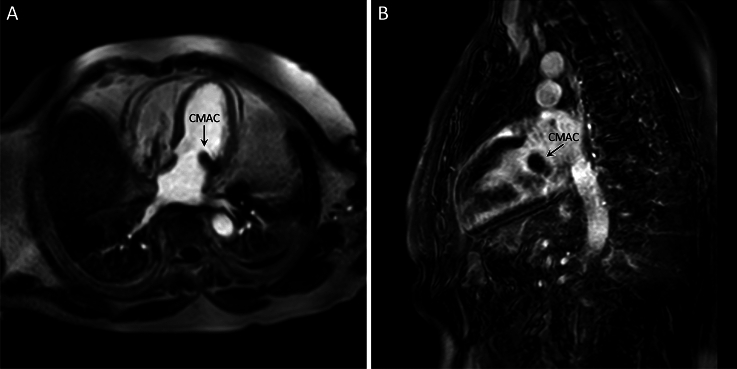
Cardiac magnetic resonance revealing a T1- and T2-weighted hypointense, calcified caseous formation of the mitral valve annulus, without early or late contrast enhancement. *CMAC* caseous mitral annular calcification

Other potential sources of coronary embolism, such as infective endocarditis and left atrial appendage thrombus have been excluded by TEE. Furthermore, no atrial fibrillation or flutter was detectable during prolonged ECG Holter monitoring.

CMAC is a rare variant of the mitral annular calcification, a chronic degenerative process of the fibrous mitral annulus that occurs at advanced age [1–3]. Histopathologically, CMAC is characterized by a liquefaction necrosis of the central region with amorphous eosinophilic acellular material, that is surrounded by macrophages and lymphocytes and bordered by multiple calcifications [4]. Although secondary consequences such as mitral valve regurgitation or stenosis caused by CMAC have been reported, a conservative approach is generally recommended due to the general benign prognosis [5–7]. However, patients with CMAC are at increased risk of arterial thromboembolism, especially embolic strokes, that are not related to atrial fibrillation [8]. The suspected pathomechanism of systemic embolism include embolization of calcium and cholesterol particles, surface ulceration with subsequent thrombus formation and embolization as well as fistulation of caseous necrotic material into the left atrium or ventricle (8, 9).

The reported case demonstrates the potentially life-threatening complication that may arise from CMAC. Thus, close clinical follow-up with multi-center research focusing on possible consequences of CMAC and a critical evaluation for oral anticoagulation is warranted. In the present case, the heart team recommended surgical resection of the tumor, which the patient declined. Therefore, oral anticoagulation with a non-vitamin K agent was initiated to prevent further thromboembolic events.

### Supplementary Information

Below is the link to the electronic supplementary material.

Supplementary file1 (MP4 5423 KB) Transesophageal echocardiography acquisition of caseous mitral annular calcification with mitral regurgitation

Supplementary file2 (MP4 3710 KB) Transesophageal echocardiography acquisition of caseous mitral annular calcification with mitral regurgitation

Supplementary file3 (MP4 2195 KB) Transesophageal echocardiography acquisition of caseous mitral annular calcification with mitral regurgitation

Supplementary file4 (MP4 2584 KB) Transesophageal echocardiography acquisition of caseous mitral annular calcification with mitral regurgitation

Supplementary file5 (MP4 2426 KB) Coronary angiography videos of the patient including follow-up angiogram after thrombus resolution.

Supplementary file6 (MP4 2352 KB) Coronary angiography videos of the patient including follow-up angiogram after thrombus resolution.

Supplementary file7 (MP4 2689 KB) Coronary angiography videos of the patient including follow-up angiogram after thrombus resolution.

## Data Availability

The data underlying this article will be shared on reasonable request to the corresponding author.
